# A case report on incidentally detected pulmonary sclerosing pneumocytoma: a diagnostic challenge

**DOI:** 10.1097/MS9.0000000000002481

**Published:** 2024-08-14

**Authors:** Golam Mursalin, Mehede H. Sawon, Md. Kamrul Alam, Salwa Islam

**Affiliations:** a Department of Thoracic Surgery, Dhaka Medical College and Hospital; b Pi Research and Development Center, Dhaka, Bangladesh

**Keywords:** case report, computed tomography, fine-needle aspiration cytology, histopathological findings, pulmonary sclerosing pneumocytoma

## Abstract

**Introduction and importance:**

Pulmonary sclerosing pneumocytoma (PSP) is a rare non-cancerous lung tumor that is usually asymptomatic, but may cause respiratory distress if it becomes large. PSPs are often detected incidentally because of their slow growth, lack of symptoms, characteristic radiographic features, and increased use of imaging studies. Although it is not a malignant tumor, it can mimic malignancy on imaging and histology, leading to misdiagnosis and unnecessary surgery.

**Case presentation:**

A 23-year-old asymptomatic female was incidentally diagnosed with PSP during evaluation for a breast fibroadenoma. A chest CT revealed a 3 cm lobulated mass in the left upper lobe. Cytology showed malignant cells with necrotic debris. Immunohistochemistry was positive for TTF-1 and EMA, negative for p63 and AE1/AE3. Histopathology confirmed a well-circumscribed benign neoplasm, consistent with pulmonary sclerosing pneumocytoma. There was no mediastinal lymph node invasion, and the post-surgery prognosis was good.

**Clinical discussion:**

PSP is a slow-growing tumor that is often asymptomatic until it reaches a significant size. Owing to their well-circumscribed margins and the presence of calcifications, they are often detected incidentally during imaging studies, such as routine chest radiography or CT scans for unrelated conditions. Although these tumors are often incidental, it is important to diagnose and treat them appropriately to prevent potential complications and malignant transformation.

**Conclusion:**

The findings of this study contribute to the existing literature, increase awareness of this rare tumor, and provide insights into its diagnosis, treatment, and follow-up.

## Introduction

Highlights

- Pulmonary sclerosing pneumocytoma (PSP) is a rare benign lung tumor that is usually asymptomatic and detected incidentally.
- When enlarged, pulmonary sclerosing pneumocytoma may cause respiratory distress.
- Pulmonary sclerosing pneumocytoma is uncommon in young females, as it predominantly affects elderly women.
- Management of pulmonary sclerosing pneumocytoma challenging because of nonspecific radiological characteristics that mimic malignancies and their histological heterogeneity.

The rare benign lung tumor known as pulmonary sclerosing pneumocytoma (PSP) was first identified more than 50 years ago by Liebow and Hubbell as a pulmonary sclerosing hemangioma^1^. The nomenclature of this tumor was changed to sclerosing pneumocytoma in 2015, and it is now included in the adenoma group according to the most recent WHO classification^2^. PSP primarily affects Asian middle-aged individuals over 50 years, and females are affected five times more frequently than males^3,4^. Although there are no conclusive diagnostic radiologic findings, it typically appears on imaging as a single, well-circumscribed nodule or mass. PSP is histologically composed of round stromal cells and cuboidal surface cells, both of which are thought to be cancerous^4^. Owing to overlapping radiologic and histologic characteristics, a diagnostic dilemma may arise between sclerosing pneumocytoma and lung adenocarcinoma^5^. Here, we report a case of surgically diagnosed PSP in a young female and discuss the challenges encountered during diagnostic evaluation and treatment. The case report has been reported in line with the SCARE criteria^6^.

## Presentation of the case

A 23-year-old woman presented for an assessment of a fibroadenoma in her right breast. During this workup, a chest X-ray incidentally revealed a solitary pulmonary nodule in the upper lobe of her left lung. She had no history of smoking, tuberculosis, or exposure to individuals with tuberculosis. Her family history did not reveal any significant lung disease or lung cancer. She had no complaints of cough, hemoptysis, dyspnea, fever, chills, night sweats, or weight loss.

**Figure 1:**
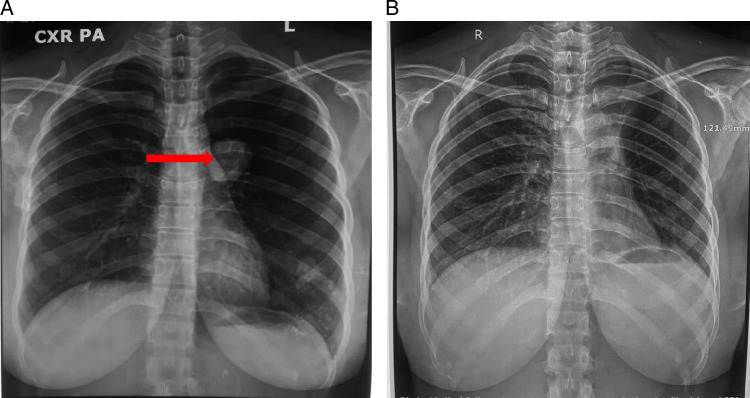
(A) Pre-operative radiograph showing a well-circumscribed solitary nodule in upper left lobe of lung. (B) Post-operative radiograph showing no traces of the nodule in upper left lobe of lung.

**Figure 2:**
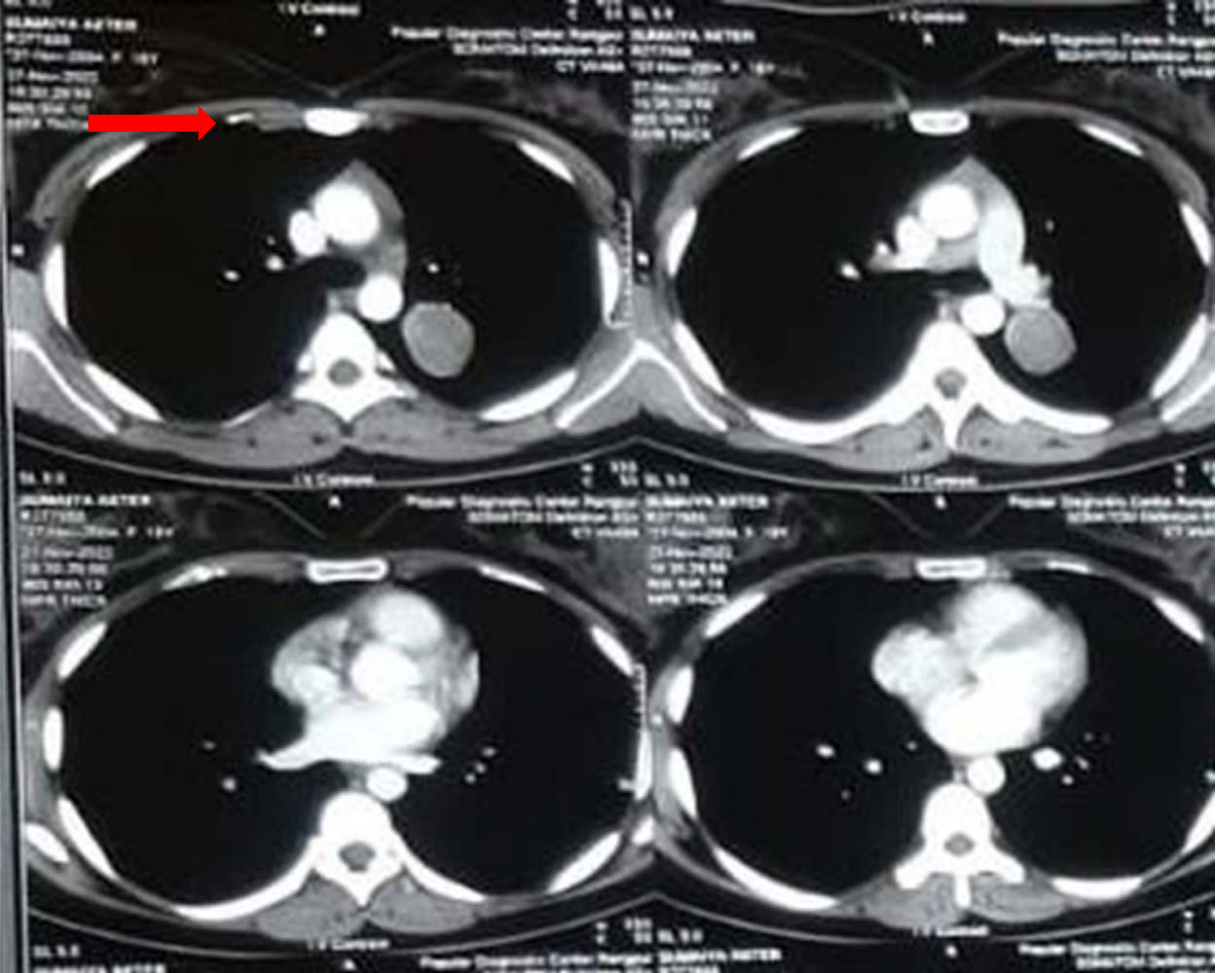
Contrast-enhanced chest CT scan showing a soft-tissue lesion ~3 cm in diameter (red arrow) located in the upper left lobe near the hilar region.

Initial chest X-ray revealed a well-circumscribed solitary nodule located in the upper lobe of the left lung (Fig. 1A). A contrast-enhanced chest computed tomography (CT) scan was then performed, which confirmed the presence of a clearly defined lobulated soft-tissue mass, which appeared less dense than the surrounding tissue (hypodense). The mass measured ~3 cm in diameter and was situated in the left upper lobe near the hilar region (Fig. 2). Laboratory tests revealed B-HCG, serum alpha-fetoprotein (AFP), lactate dehydrogenase (LDH), carcinoembryonic antigen (CEA), and cancer antigen (CA) 19-9 levels were within normal ranges. Mediastinal lymphadenopathy was not observed.

Subsequently, the patient underwent bronchoscopy, CT-guided fine-needle aspiration cytology (FNAC), and immunohistochemistry (IHC) analysis. The bronchoscopy indicated a normal appearance of the patient’s endobronchial tree, while FNAC of the mass identified malignant tumor cells amid necrotic material. Immunohistochemistry testing showed positive staining for thyroid transcription factor-1 (TTF-1), with negative staining for pulmonary endocrine tumors (p63), ruling out a diagnosis of carcinoid tumor. Malignancy was suspected, but there was no indication of the cancer spreading to nearby or distant regions; therefore, the patient underwent left upper lobectomy along with mediastinal lymph node sampling, and the specimens were sent for histopathological examination.

Histopathological examination of the specimen showed solid and nodular growth with a maximum diameter of 3.2 cm. The growth was well-defined with a variegated appearance, displaying colors ranging from tan-gray to brownish. Microscopic findings demonstrated a well-circumscribed non-capsulated benign neoplasm, composed of cuboidal cells lining spaces containing RBCs and papillae, as well as round to polygonal stromal cells in sheets and trabeculae. Aggregates of histiocytes, chronic inflammatory cell infiltration, and fibrosis were also observed. IHC findings showed TTF-1 and EMA positivity and pancytokeratin (AE1/AE3) negativity in tumor cells. Reactive hyperplasia was observed in the lymph nodes. The histological and IHC findings were in line with the diagnosis of sclerosing pneumocytoma; there was no indication of invasion of the mediastinal lymph node, and the prognosis after surgery was favorable.

## Discussion

Pulmonary sclerosing pneumocytoma (PSP) is a slow-growing tumor that is often asymptomatic until it reaches a significant size. Owing to their well-circumscribed margins and the presence of calcifications, they are often detected incidentally during imaging studies performed for other medical reasons, such as routine chest radiography or CT scans for unrelated conditions. Although these tumors are often incidental, it is important to diagnose and treat them appropriately to prevent potential complications and malignant transformation. Furthermore, distinguishing between adenocarcinoma and carcinoid tumors remains difficult because of the nonspecific radiological characteristics that mimic malignancies and their histological heterogeneity. Our patient was a 23-year-old woman with solitary PSP located in the left upper lobe near the hilar region of the lung.

The radiological results in PSP cases are generally nonspecific, and a chest CT scan with contrast enhancement typically demonstrates a well-defined solitary nodule with varying degrees and patterns. Approximately 25–59% of PSP cases have this heterogeneous internal texture and larger PSPs generally tend to show more heterogeneous enhancement^7,8^. The radiological findings reported in our study can aid in the identification of PSPs as a peripheral, solitary, well-defined mass. However, PSPs are often situated in the lower lobes, with nearby vessels in a significant proportion of cases (26.3%)^7^; however, in our case, PSP was located in the upper lobe. Similar to our case, PSPs are frequently asymptomatic and found incidentally; however, some patients may experience symptoms such as cough, hemoptysis, chest pain, or shortness of breath^3^. In rare circumstances, regional lymph node metastasis may be observed, but this does not affect the prognosis^7^. Since these features are not specific to sclerosing pneumocytoma and can also be observed in other lung tumors, histological examination is necessary for a definitive diagnosis^3^.

When feasible, preoperative diagnosis is useful for determining and planning the best therapeutic strategy. The presence of cuboidal surface cells and round stromal cells, which are found in four major histological patterns (solid, papillary, sclerotic, and hemangiomatous), is a key characteristic of PSP^2^. Cytological features correspond to these histological patterns, with at least two of the four architectural patterns, typically papillary and solid patterns, present in all patients, and three patterns in 95% of patients^9,10^. Gal *et al*. observed that identifying PSP’s dual cell population, with many stromal cells and few surface cells, is essential for cytological diagnosis of the disease^11^.

When plentiful histocytes, foamy macrophages, and respiratory ciliated cells are observed, the background, which is frequently hemorrhagic and without necrosis, may also help with the diagnosis. However, in our case, necrotic debris was seen in the background, which made it difficult to rule out carcinoid tumors. Because the disease is uncommon and most pathologists are unfamiliar with its cytological features, the diagnostic performance of fine-needle aspiration (FNA) cytology is poor. This is because the two tumor cell types were rarely clearly separated in the specimen. Few case reports have described the preoperative cytological and histological features of PSP, and these publications frequently misdiagnosed patients using intraoperative frozen sections (FS), endobronchial ultrasound-guided transbronchial needle aspiration (EBUS-TBNA)^11,12^, or CT-guided FNA^4,13–16^. Identifying this type of tumor is difficult because of its varied histological features. Fine-needle aspiration (FNA) cytology can result in varying smear samples, ranging from hypocellular, bloody, sclerotic to hypercellular, loaded with stromal fragments, and/or exhibiting the growth of epithelial cells^13^. The type of smear obtained during needle biopsy sampling depends on the location, making it crucial to obtain representative material in the cell block to identify different cell patterns and perform immunohistochemistry for accurate diagnosis^10,14,17^.

The presence of two distinct cell populations in PSPs results in a distinct immunohistochemical profile that aids in distinguishing and differentiating between the two populations. Both cell types, surface/papillary cells and round stromal cells, stained positive for TTF-1 and EMA, and negative for pulmonary endocrine tumors (p63). However, the surface cells stained positive for pancytokeratin (AE1/AE3), and the round stromal cells stained negative for pancytokeratin (AE1/AE3). This prompted us to assume that the sclerosing pattern might indicate the neoplasm’s regressive characteristics, whereas the papillary pattern represents its benign component. Conversely, the solid growth pattern indicates the presence of hormone-related factors that stimulate higher rates of cell division, leading to the development of neoplastic growth. This can contribute to the development of cancerous growth and explain why these tumors, despite having a relatively low likelihood, are still capable of becoming malignant^11^.

Breast cancer and PSP developing simultaneously in our instance are rare events that are frequently regarded as coincidental in the literature^11^. Distinguishing between PSP and breast cancer metastases, given their comparable radiological features, is challenging due to their synchronous existence. In this situation, cases with minimal or no atypia should always have PSP staining for TTF-1 along with estrogen receptors (ER) and progesterone receptors (PR) included in the immunohistochemical analysis^18^. Similar to our case, relying only on TTF-1 staining for diagnosis is insufficient because it can lead to misdiagnosis due to its lack of specificity, and TTF-1 staining can be found in primary adenocarcinomas, metastatic thyroid carcinomas, and carcinoid tumors^10^.

## Conclusion

The challenge in identifying this uncommon tumor is highlighted in this case report of PSP. Even if preoperative FNAC shows primary malignancy, clinicians need to be aware of the risk of false-positive results for solitary lung nodules that grow slowly and are more than 2 cm in size. It is essential for both surgeons and pathologists to have knowledge of this rare type of tumor. However, diagnosing this type of tumor is difficult because of limited familiarity with its histological and immunohistopathological features. Adequate specimen size is essential for obtaining representative material in the cell block, which is crucial for pre- and intraoperative diagnosis and plays a vital role in guiding surgical decision-making.

## Ethical approval

No ethical approval was obtained because this was a single case report emerging during routine practice. The authors confirm that no harm was caused to human subjects and that the study procedures were in compliance with the ethical standards and regulations established by the World Medical Association’s Helsinki Declaration 2013.

## Consent

Written informed consent was obtained from the patient for publication of this case report and accompanying images. A copy of the written consent is available for review by the Editor-in-Chief of this journal on request.

## Source of funding

The authors have no support or funding to report.

## Author contribution

G.M., M.H.S., M.K.A., and S.I.: study conception and design, data analysis, and interpretation; G.M., M.H.S., M.K.A., and S.I.: drafting the manuscript; G.M., M.H.S., M.K.A., and S.I.: data acquisition, data interpretation, and critical revision of the manuscript for important intellectual content. All authors approved the final manuscript.

## Conflicts of interest disclosure

All authors declare that there are no conflicts of interest regarding the publication of this paper.

## Research registration unique identifying number (UIN)

Not applicable.

## Guarantor

Golam Mursalin, Mehede Hasan Sawon, Md. Kamrul Alam, and Salwa Islam.

## Data availability statement

Data and materials pertaining to individual patients can be accessed upon request from the corresponding author.

## Provenance and peer review

Not applicable.
